# Biomechanical evaluation of aortic regurgitation from cusp prolapse using an ex vivo 3D-printed commissure geometric alignment device

**DOI:** 10.1186/s13019-022-02049-5

**Published:** 2022-12-10

**Authors:** Yuanjia Zhu, Matthew H. Park, Annabel Imbrie-Moore, Robert Wilkerson, Sarah Madira, Y. Joseph Woo

**Affiliations:** 1 Department of Cardiothoracic Surgery, Stanford University School of Medicine, 300 Pasteur Drive, Falk Cardiovascular Research Center, Stanford, CA 94305 USA; 2 Department of Bioengineering, Stanford University, Stanford, USA; 3 Department of Mechanical Engineering, Stanford University, Stanford, USA

**Keywords:** Aortic cusp prolapse, Aortic commissure, Commissure height, Inter-commissure angle, Aortic regurgitation

## Abstract

**Background:**

Aortic regurgitation (AR) is one of the most common cardiac valvular diseases, and it is frequently caused by cusp prolapse. However, the precise relationship of commissure position and aortic cusp prolapse with AR is not fully understood. In this study, we developed a 3D-printed commissure geometric alignment device to investigate the effect of commissure height and inter-commissure angle on AR and aortic cusp prolapse.

**Methods:**

Three porcine aortic valves were explanted from hearts obtained from a meat abattoir and were mounted in the commissure geometric alignment device. Nine commissure configurations were tested for each specimen, exploring independent and concurrent effects of commissure height and inter-commissure angle change on AR and aortic cusp prolapse. Each commissure configuration was tested in our 3D printed ex vivo left heart simulator. Hemodynamics data, echocardiography, and high-speed videography were obtained.

**Results:**

AR due to aortic cusp prolapse was successfully generated using our commissure geometric alignment device. Mean aortic regurgitation fraction measured for the baseline, high commissure, low commissure, high commissure and wide inter-commissure angle, high commissure and narrow inter-commissure angle, low commissure and wide inter-commissure angle, low commissure and narrow inter-commissure angle, wide commissure, and narrow commissure configurations from all samples were 4.6 ± 1.4%, 9.7 ± 3.7%, 4.2 ± 0.5%, 11.7 ± 5.8%, 13.0 ± 8.5%, 4.8 ± 0.9%, 7.3 ± 1.7%, 5.1 ± 1.2%, and 7.1 ± 3.1%, respectively.

**Conclusions:**

AR was most prominent when commissure heights were changed from their native levels with concomitant reduced inter-commissure angle. Findings from this study provide important evidence demonstrating the relationship between commissure position and aortic cusp prolapse and may have a significant impact on patient outcomes after surgical repair of aortic valves.

**Supplementary Information:**

The online version contains supplementary material available at 10.1186/s13019-022-02049-5.

## Background

Aortic regurgitation (AR) is one of the most common cardiac valvular diseases, affecting up to 13% of the population in the United States [1]. The pathophysiology of AR can be classified based on its function. AR can be due to functional aortic annulus dilation, cusp perforation, or restriction [2]. Type II AR, which is caused by cusp prolapse due to excess cusp tissue or commissural disruption [2], is another important class of AR because the aortic cusp tissue is otherwise normal. Aortic cusp prolapse can be corrected with a variety of aortic repair techniques, such as free margin plication and resuspension [3]. Our group has previously designed several ex vivo AR models using porcine and bovine aortic valves, and we have found that aortic commissure position play a critical role in supporting the proper geometry of aortic cusps and the function of the aortic root apparatus [4–6]. In particular, both commissure height relative to the annulus and inter-commissure angle can affect aortic cusp position and impact coaptation. This observation correlates well with the clinical finding that low commissure height was frequently associated with AR recurrence from cusp prolapse after valve reimplantation repair [7, 8]. Despite these general findings, the precise relationship of commissure position, both in height and inter-commissure angle, and AR due to aortic cusp prolapse is not fully understood. As such, early failure after valve repair still occurs, and long-term repair durability can still be improved. In this study, we developed a 3D-printed commissure geometric alignment device that allows for precise, simultaneous manipulation of commissure height and inter-commissure angle. Using our 3D printed ex vivo left heart simulator, we aim to elucidate the impact of commissural misalignment on AR and cusp prolapse. [4–6, 9]

## Methods

### Commissure geometric alignment device design

**Fig. 1:**
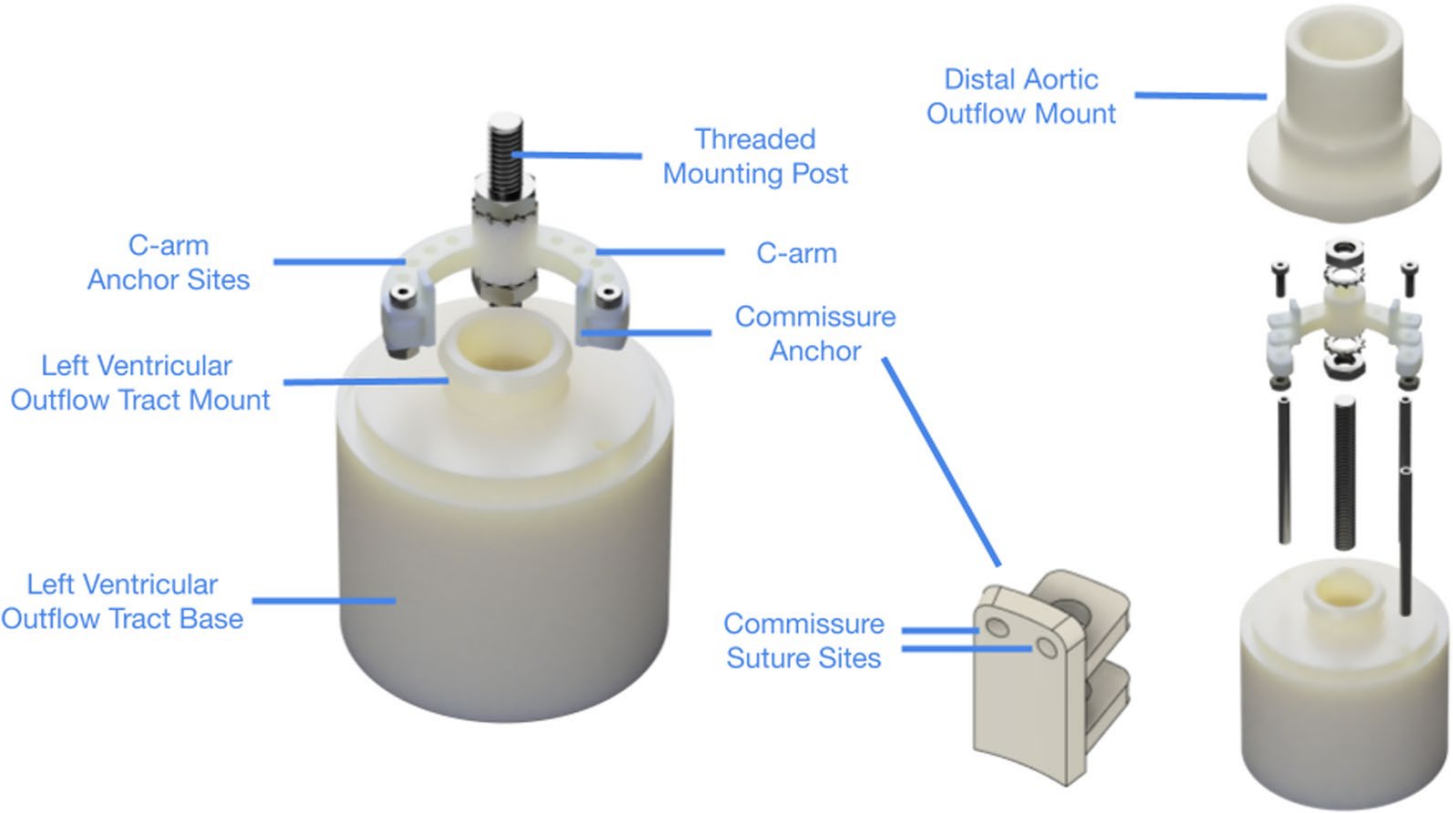
Computer aided design renderings of the commissure geometric alignment device. This device contains the left ventricular outflow tract base, the left ventricular outflow tract mount, the distal aortic outflow mount, and a threaded mounting post where a custom-sized c-arm is attached. The position of the c-arm can be adjusted along the height of the anchor, allowing for commissure height adjustment. Along the c-arm, multiple anchor sites were designed such that commissure anchors can be secured onto the c-arm to adjust the inter-commissure angle

A 3D-printed commissure positioning device was designed to independently manipulate any two of the commissures of an explanted aortic valve (Fig. 1). This device contains three 3D-printed components from our baseline aortic mount previously described for aortic valve simulation studies: the left ventricular outflow tract (LVOT) base, the distal aortic outflow mount, and the posts aligning the base and the distal mount [4–6, 9]. To the LVOT base, an additional threaded mounting post was added with a custom-sized, 3D-printed c-arm that was secured using lock washers and hex nuts. The position of the c-arm can be adjusted and secured along the height of the anchor, allowing for commissure height adjustment. The diameter and arc length of the c-arm was custom designed to fit the aortic dimension of each specimen to minimize radial commissural distortion. Commissure anchors were designed to be sutured to the aortic commissures of the explanted valve and to be fixed to the c-arm for precise commissure angle positioning. Each anchor has two suture sites or small perforations to allow suture attachment of the commissure onto the anchor. Along the c-arm, multiple anchor sites or holes were added such that the commissure anchors could be secured via screws onto the c-arm to adjust the inter-commissure angle. The inter-commissure angle change between the two adjacent anchor sites along the c-arm is 15 degrees. Therefore, by adjusting the c-arm height and commissure anchor positions, the commissure position can be manipulated with two degrees of freedom. The maximum height adjustment of this system is 18.325 mm, and the maximum inter-commissure angle adjustment is 180°.

### Sample preparation

**Fig. 2:**
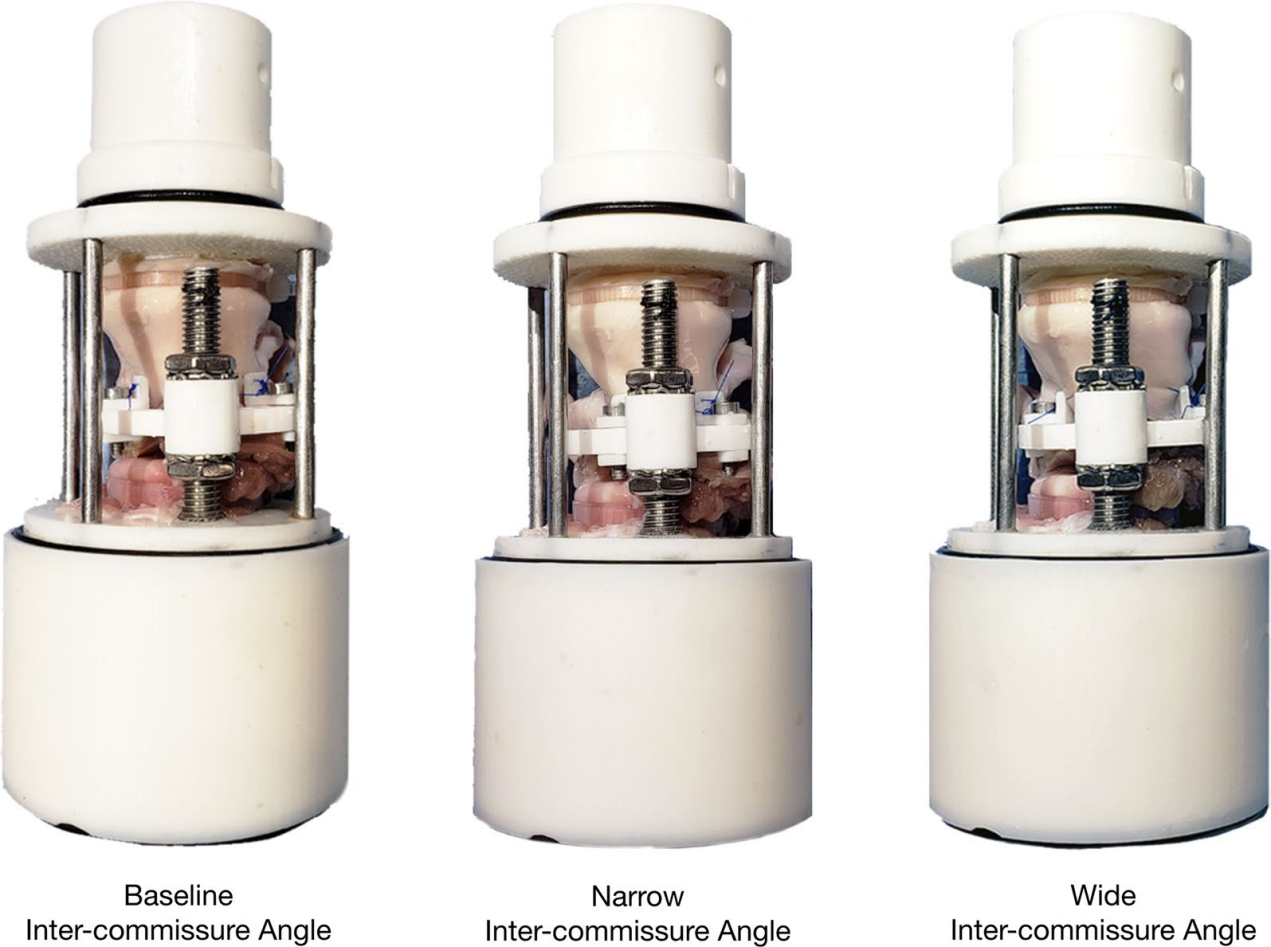
Porcine aortic valves mounted onto the commissure geometric alignment device. Note the difference in the inter-commissure angle generated using the same specimen

Porcine aortic valves (n = 3) were harvested from hearts obtained from a meat abattoir. The aortic valves were carefully explanted to preserve 1 cm of the LVOT, the annulus, aortic cusps, left and right coronary arteries, and ascending aorta. The distal LVOT was trimmed to minimize excess tissue. The aortic valve specimen was oriented such that the non-coronary cusp was facing the c-arm anchor. The distal LVOT was then secured onto the LVOT mount using a cable tie. 4–0 polypropylene sutures were used to attach the left-noncoronary commissure and the right-noncoronary commissure onto commissure anchors. The c-arm height was adjusted to the same level of the native commissure height and secured via lock washers and nuts. The commissure anchors were secured in the corresponding c-arm anchor sites to maintain the native geometry at an approximately 120° angle. The distal aorta was finally attached to the distal aortic outflow mount using a cable tie. This completes the baseline native commissure configuration (Fig. 2).

**Fig. 3:**
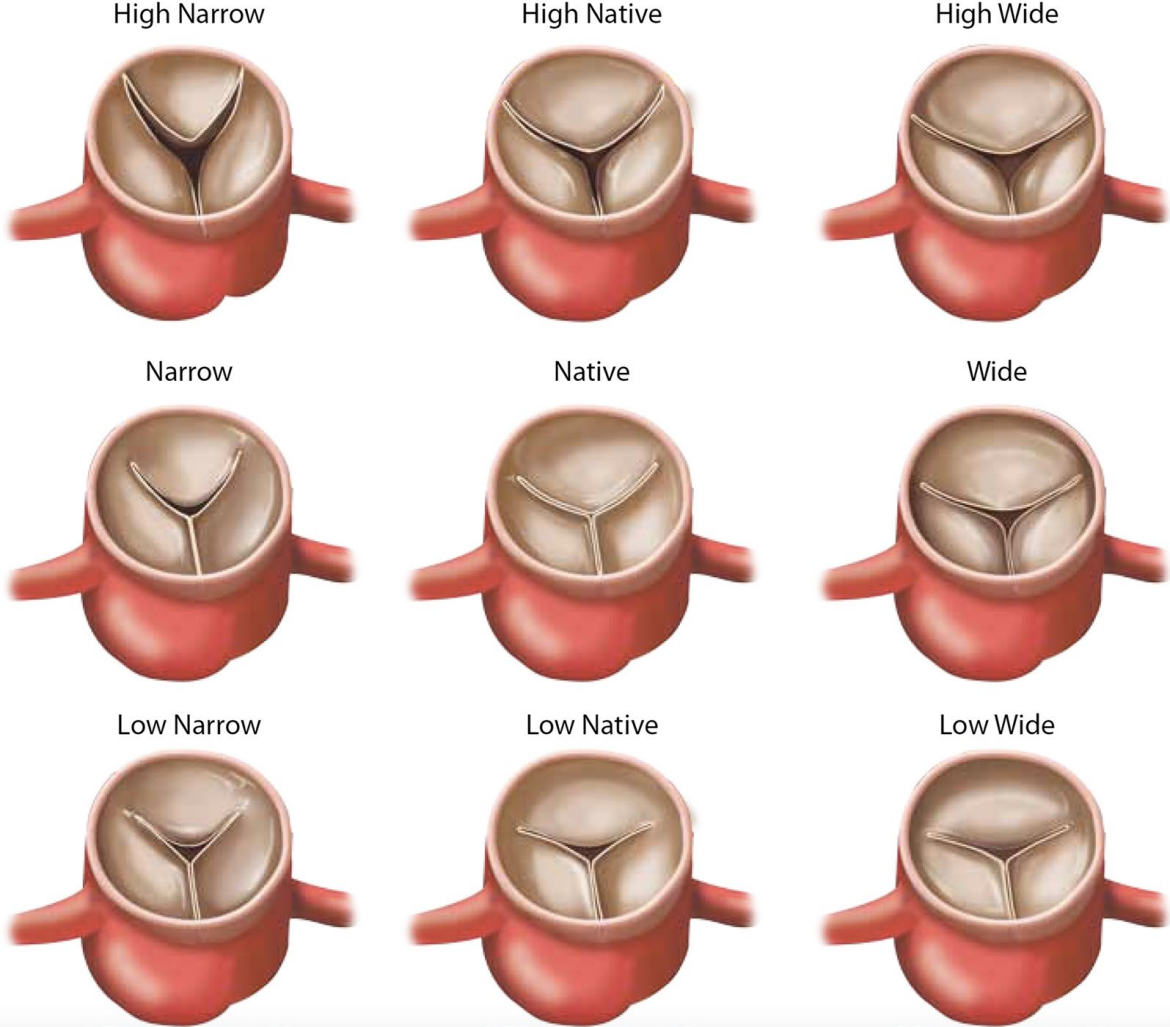
An illustration of the nine commissure configurations by varying the commissure height and the inter-commissure angle. The associated cusp prolapse phenotypes were also demonstrated

To evaluate the relationship of commissure position and AR due to aortic cusp prolapse, 8 other commissure configurations were tested. These configurations were generated by additional commissure height and angle adjustments (Fig. 3), namely: high commissure (H), low commissure (L), wide inter-commissure angle (W), narrow inter-commissure angle (N), high commissure and wide inter-commissure angle (HW), high commissure and narrow inter-commissure angle (HN), low commissure and wide inter-commissure angle (LW), and low commissure and narrow inter-commissure angle (LN). To generate the high commissure configuration, the c-arm was moved upwards by 9 mm without adjusting the positions of the commissure anchors. Conversely, to create the low commissure configuration, the c-arm was moved downwards by 9 mm without moving the commissure anchors. To generate the narrow inter-commissure angle configuration, the two commissure anchors were moved towards each other by 15° each and resecured in the c-arm with screws (Fig. 2). Similarly, to create the wide inter-commissure angle configuration, the two commissure anchors were moved away from each other by 15° each and secured in the c-arm (Fig. 2). The other combinational configurations were achieved by manipulating the c-arm and the commissure anchors simultaneously in the same fashion described above. When commissure angle was changed from the native angle, the LVOT cable tie was removed to allow the LVOT tissue to adapt to the new inter-commissure angle, and then the LVOT was re-attached. Similarly, the distal aortic tissue was removed from the distal aortic outflow mount, readjusted, and re-attached to the outflow mount in the same fashion described above. Each specimen served as its own control using the baseline configuration. The same specimens were re-tested in all 8 other configurations defined above.

### Left heart simulator

The 3D-printed ex vivo left heart simulator has been previously described in detail [4–6, 9]. In brief, the simulator contains a programmable pulsatile linear piston pump (ViVitro Superpump, ViVitro Labs, Victoria, BC, Canada) and a viscoelastic impedance adapter (ViVitro) that is comprised of two compliance chambers and a fixed resistance element (200 dyne·s/cm^5^) and generates a physiologic ventricular waveform. An additional aortic compliance chamber is also included to allow for peripheral resistance adjustment. The system compliance and peripheral resistance are titrated to generate physiologic systolic and diastolic arterial pressures. Pressure transducers (Utah Medical Products Inc., Midvale, Utah) and electromagnetic flow probes (Carolina Medical Electronics, East Bend, North Carolina) are used to record aortic, left ventricular, and left atrial pressure and flow throughout a complete cardiac cycle. Prior to a simulation run, the simulator is calibrated using a mechanical aortic valve to obtain an effective stroke volume of 70 mL by setting the pump stroke volume to 100 mL at 70 beats per minute. The hemodynamics data for our experiments were collected and averaged across ten cycles for each commissure configuration. To evaluate leaflet morphology and motion, a high-speed videography at 1057 frames per second with 1280 × 1024 resolution (Chronos 1.4, Kron Technologies, Burnaby, British Columbia, Canada) was obtained from an *en face* view.

### Echocardiography measurements

Echocardiographic data was obtained using a Phillips iE33 system with the S5-1 transthoracic probe (Koninklijke Philips NV, Amsterdam, The Netherlands) for short- and long-axis views and color flow mappings. Continuous-wave doppler was also recorded. Data analysis was performed using the iE33 on-board software and a Siemens Syngo Dynamics workstation (Siemens Medical Solutions USA, Inc., Ann Arbor, MI).

### Statistical analysis

A two-sampled paired t-test was performed to compare the baseline commissure configuration to all other configurations. Continuous variables are reported as mean ± standard deviation. Statistical significance was defined at *p* < 0.05. Approval by the Institutional Animal Care and Use Committee was not indicated for this study as the porcine hearts were purchased from a meat abattoir.

## Results

**Fig. 4:**
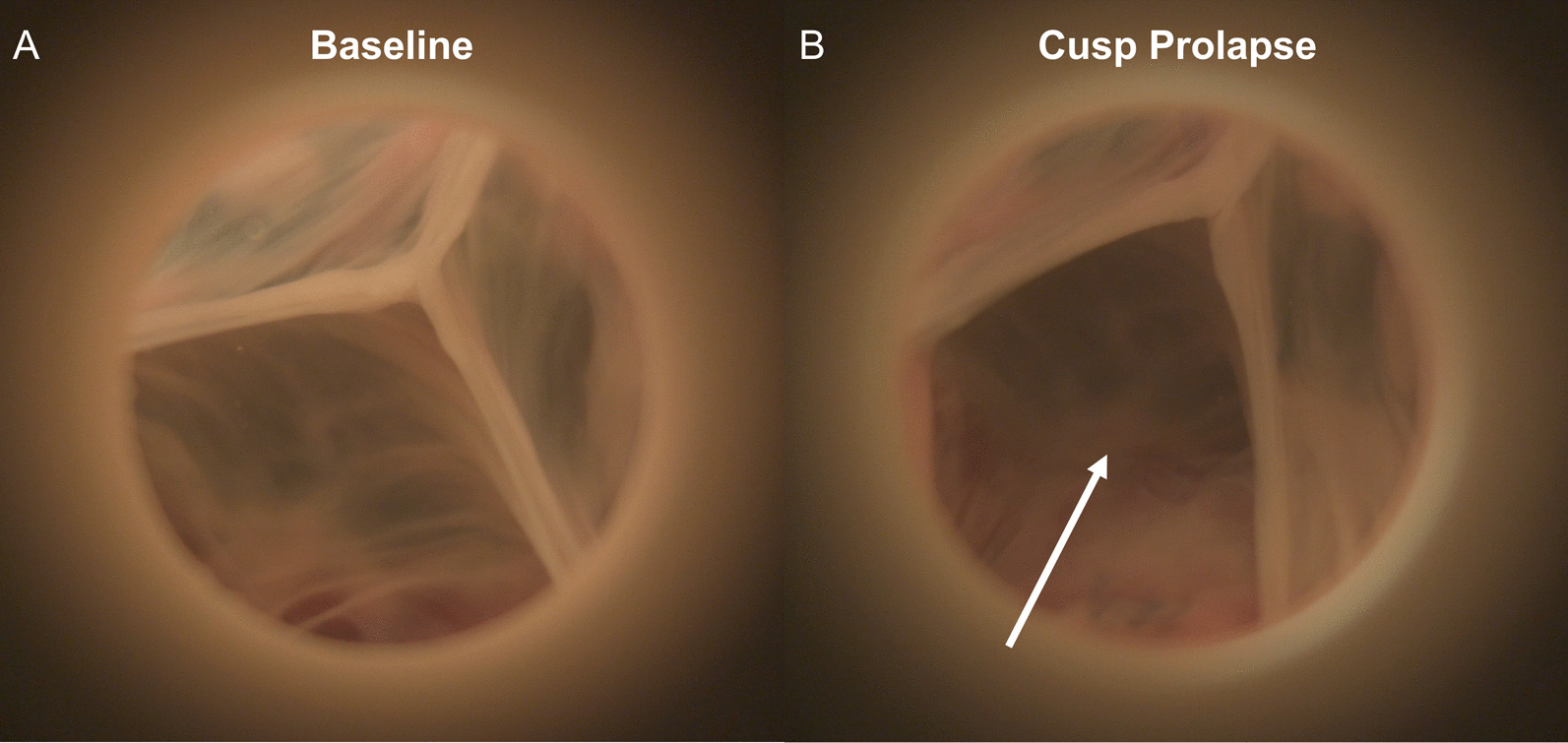
**A** Baseline porcine aortic valve mounted in the ex vivo left heart simulator as visualized in high-speed videography in diastole. **B** The same aortic valve after commissure position changes using the commissure geometric alignment device to increase the commissure height while decreasing the inter-commissure angle

**Fig. 5:**
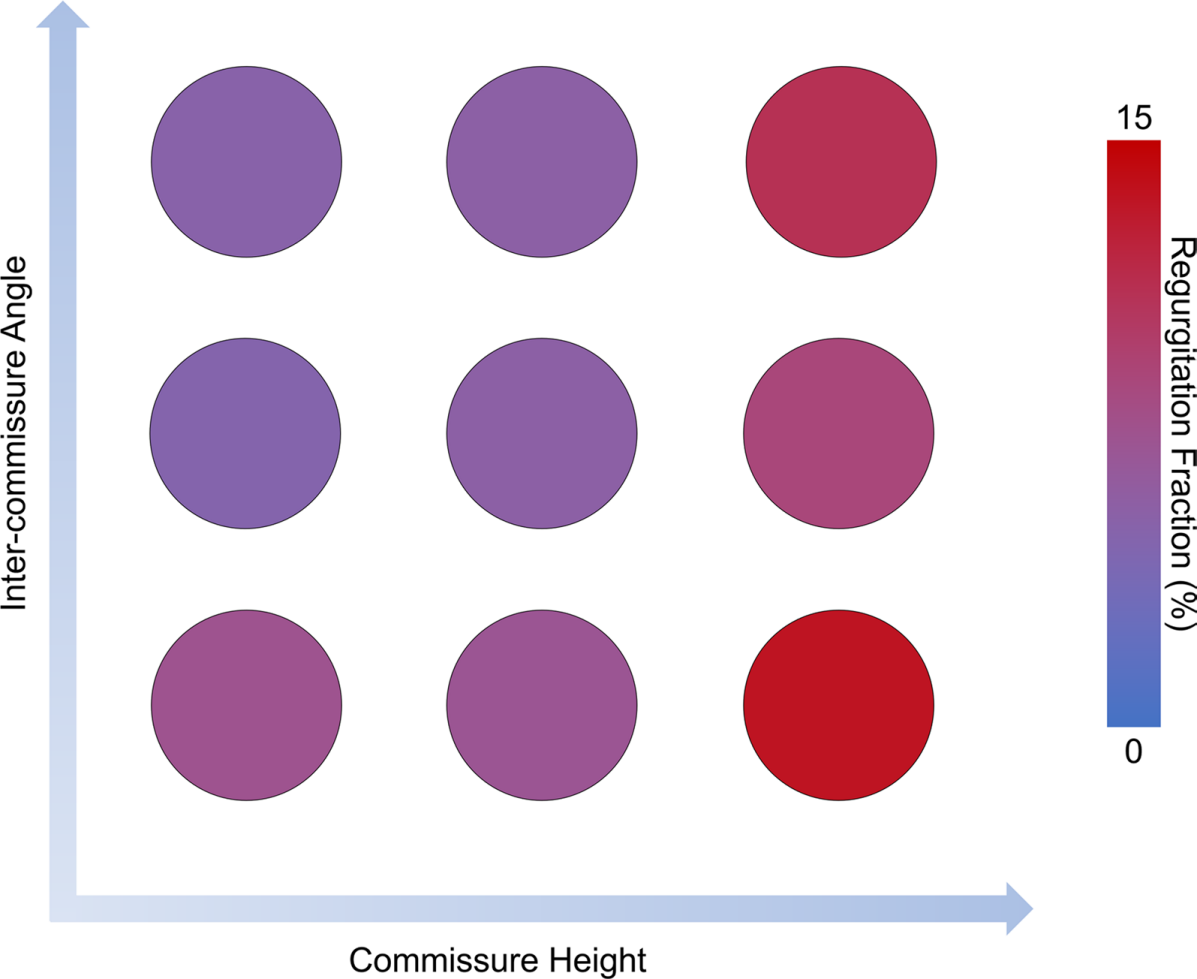
A heat map illustrating the degree of mean aortic regurgitation fraction based on commissure height and inter-commissure angle. Blue indicates no aortic regurgitation, whereas red indicates 15% aortic regurgitation measured using the ex vivo left heart simulator

AR due to aortic cusp prolapse was successfully generated using our commissure geometric alignment device as shown by the high-speed videography during diastole (Fig. 4). Specifically, aortic cusp prolapse associated AR was most notable when the inter-commissure angle was decreased while the commissure height was changed from baseline (Fig. 5). As shown in Fig. 5, changing only the commissure height or the inter-commissure angle did not significantly affect AR. However, when the commissure height is changed from the native height, the modification in inter-commissure angle could have an impact on AR, and the increase in AR was most prominent when the inter-commissure angle was decreased.

**Fig. 6:**
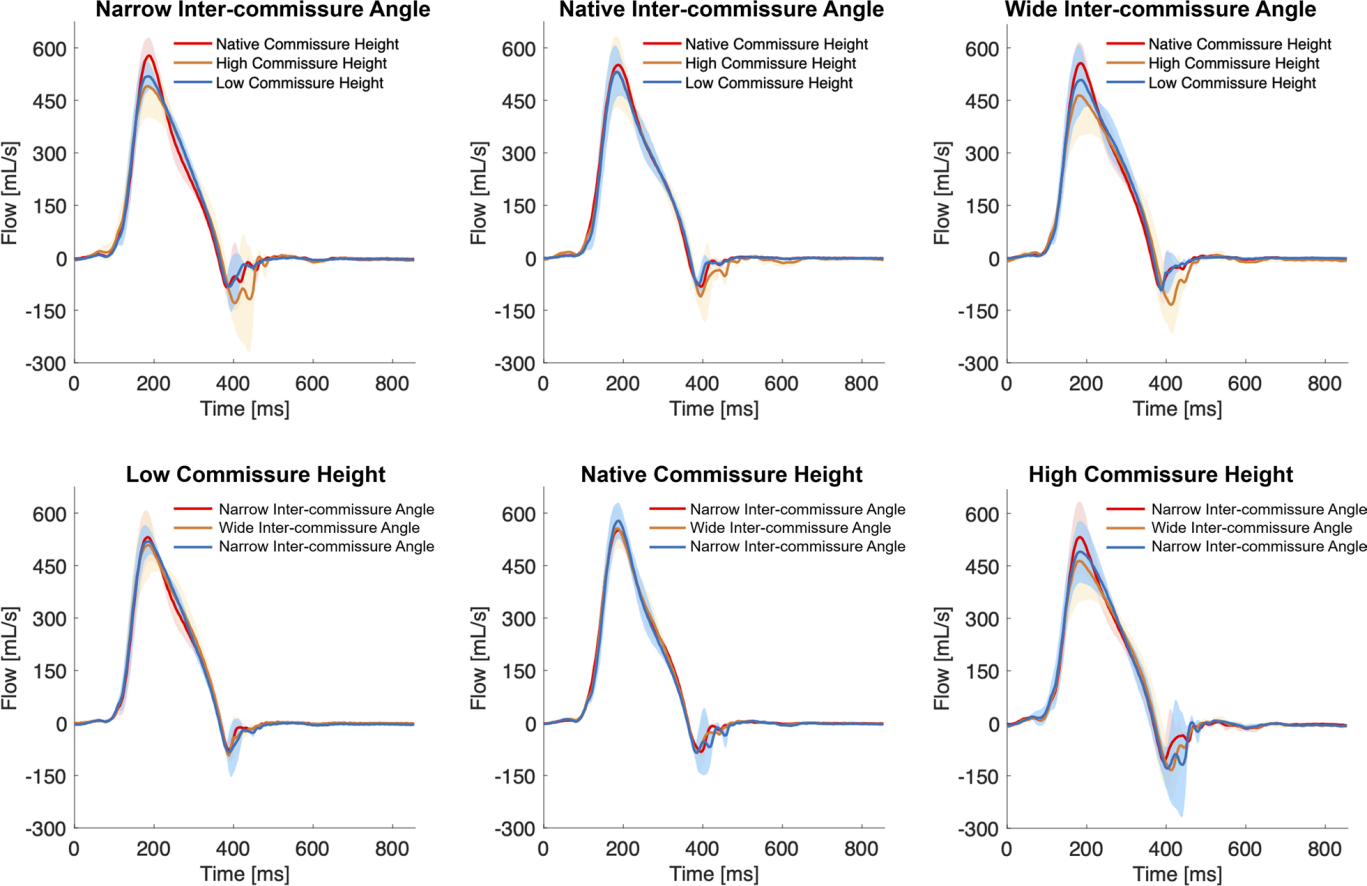
Mean aortic flow measured via the ex vivo left heart simulator of each commissure configuration. Aortic regurgitation, as evidenced by flow reversal in diastole, was most prominent when the commissure height was increased with decreased inter-commissure angle. Shaded regions represent standard deviation

**Fig. 7:**
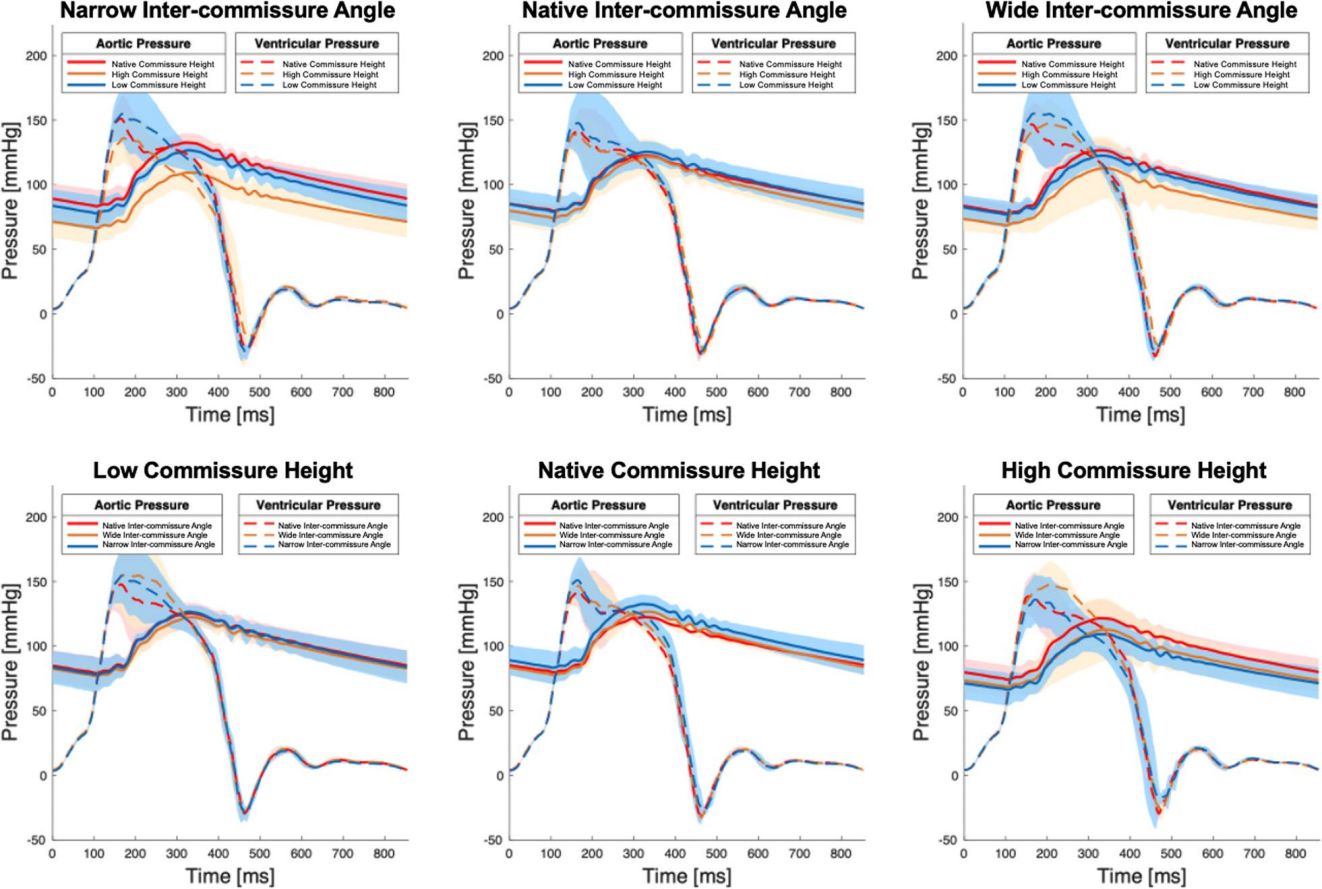
Aortic and left ventricular pressure tracings measured via the ex vivo left heart simulator of each commissure configuration. Aortic pressure tracings demonstrated lower pressures throughout the cardiac cycle when the inter-commissure angle was changed from its baseline condition and when the commissure height was increased. Shaded regions represent standard deviation

Mean aortic flow tracings and pressure tracings are shown in Figs. 6, 7. AR, as evidenced by flow reversal during diastole, was most prominent when two commissures were elevated from the native height. Both narrow and wide inter-commissure angle were shown to worsen AR, but this effect was maximized when the high commissure configuration was in place. Mean arterial pressure measured for the baseline, H, L, HW, HN, LW, LN, W, and N commissure configurations were 99.8 ± 1.0 mmHg, 96.0 ± 7.0 mmHg, 100.9 ± 7.1 mmHg, 100.6 ± 3.8 mmHg, 85.6 ± 8.7 mmHg, 97.7 ± 5.4 mmHg, 100.3 ± 6.9 mmHg, 100.6 ± 3.8 mmHg, and 106.0 ± 6.6 mmHg, respectively.

Aortic regurgitation fraction measured for the baseline, H, L, HW, HN, LW, LN, W, and N commissure configurations were 4.6 ± 1.4%, 9.7 ± 3.7%, 4.2 ± 0.5%, 11.7 ± 5.8%, 13.0 ± 8.5%, 4.8 ± 0.9%, 7.3 ± 1.7%, 5.1 ± 1.2%, and 7.1 ± 3.1%, respectively. Lastly, the aortic leakage rate measured from the baseline, H, L, HW, HN, LW, LN, W, and N commissure configurations were 1.5 ± 1.4 ml/s, 7.3 ± 4.6 ml/s, 1.9 ± 1.0 ml/s, 4.9 ± 2.3 ml/s, 5.0 ± 2.8 ml/s, 1.7 ± 3.1 ml/s, 4.9 ± 1.6 ml/s, 2.3 ± 0.8 ml/s, and 3.7 ± 0.8 ml/s, respectively. The aortic leakage rate obtained from the LN commissure configuration was significantly higher than that from the baseline configuration (*p* = 0.002). There was no difference in the aortic regurgitation fraction or aortic leakage rate between the HN and LN configurations with (*p* = 0.64 and *p* = 0.97). A summary of hemodynamics data is shown in Additional file 1: Tables S1–S3.

Additionally, transvalvular hemodynamics were measured using echocardiogram. No significant aortic stenosis was observed. The HW commissure configuration was found to have increased mean transaortic gradient compared to that of the baseline commissure configuration (*p* = 0.01). The mean gradient across the aortic valve measured for the baseline, H, L, HW, HN, LW, LN, W, and N commissure configurations were 16.3 ± 4.1 mmHg, 20.3 ± 10.7 mmHg, 14.0 ± 5.1 mmHg, 25.7 ± 10.8 mmHg, 17.7 ± 3.7 mmHg, 21.0 ± 7.5 mmHg, 21.3 ± 2.6 mmHg, 19.3 ± 5.2 mmHg, and 16.0 ± 4.5 mmHg, respectively.

## Discussion

In this study, we successfully designed a commissure geometric alignment device that can manipulate aortic commissure positions to induce aortic cusp prolapse. This device allows for simultaneous positional changes of two commissures, each with two degrees of freedom, namely height and inter-commissure angle. Aortic cusp prolapse with associated AR was most prominent when the commissure height was deviated from the baseline level with concomitant reduced inter-commissure angle. AR was confirmed through hemodynamic data. The findings from this study elucidated the relationship between aortic cusp prolapse and commissure position, specifically height and inter-commissure angle.

For the geometric alignment device, we deliberately chose to simultaneously manipulate two commissures. Since each aortic cusp is attached to the two adjacent commissures distally [10], changing the positions of these two commissures could maximally impact the aortic cusp that is attached to them without exerting significant effect on the other two aortic cusps, as the third commissure was kept at its native position.

Aortic cusp prolapse can be visualized as an aortic cusp that is below its physiologic height of coaptation and often prolapses into the left ventricle during diastole [11]. Intraoperatively, the prolapsed cusp can be identified as having excess free margin length and tissue [11, 12]. Various aortic cusp repair techniques have been described, aimed at reducing the excess free margin to re-create a proper coaptation plane for the prolapsed cusp. [2, 11, 12] Therefore, we hypothesized that to induce aortic cusp prolapse, relative excess tissue must be introduced. This can be most effectively achieved by decreasing the inter-commissure angle. However, as observed in this study, decreasing the inter-commissure angle alone was not sufficient to introduce cusp prolapse with AR. This is likely because the affected aortic cusp was still suspended by the two commissures at their native heights, allowing the cusp to coapt with the other two cusps at the same height. By adjusting the two adjacent commissure heights, the affected aortic cusp can no longer meet the other two cusps at the same plane for proper coaptation.

One interesting observation was that commissure height change alone had minimal effect on aortic cusp prolapse with AR using the commissure geometric alignment device. We have previously described an ex vivo AR model from cusp prolapse, which can be produced by detaching the two adjacent commissures from the aortic wall and reimplanting them 5 mm below the native height [6]. Note that our prior model also required a small portion of the two adjacent cusps to be detached from the aortic annulus to allow an increased degree of freedom for commissure manipulation. Although the commissure geometric alignment device was able to lower the two commissures by more than 5 mm, native aortic root anatomy was not altered surgically. Tension was observed in the aortic wall tissue as the commissure positions were changed from the native sites using the device. Furthermore, the fibrous bands on the aortic cusp tissue that help anchor the cusp to the aortic wall act as another protective mechanism to prevent cusp geometry alteration from its native state [11, 13]. These anatomic details were not altered by the device and can affect aortic cusp motion and function.

Nonetheless, findings from this study provided important insights into aortic cusp anatomy and geometry as well as their effect on cusp prolapse with AR. Specifically, results from this study can be directly translated to valve reimplantation procedures, such as valve-sparing aortic root replacement (VSRR), that are used to address aortic root aneurysm with or without AR [14–16]. As the VSRR procedure requires re-implanting the aortic commissures into the Dacron aortic graft [14, 16], care must be taken to optimize the commissure positions to recreate proper aortic cusp coaptation planes. However, limited information is available regarding the precise commissure location and aortic root geometry required to minimize AR. As a result, postoperative AR after VSRR is unfortunately not uncommon [17, 18]. Besides annular dilation leading to central AR, aortic cusp prolapse is another important mode of repair failure [17]. Several clinical papers have emphasized the importance of re-creating proper cusp coaptation and geometry during the operation [19–23]. Specifically, cusp coaptation below the level of the aortic annulus has been found to be associated with AR progression [23]. Additionally, adequate coaptation lengths and effective heights should be achieved intraoperatively to decrease the risk of AR occurrence after the repair [19, 21]. This study suggests that when suspending the commissures, care should be taken to optimize commissure height, especially when perfectly symmetric commissure angles cannot be ensured in the operating room due to a lack of Dacron grafts with 120° markings. Together with the important insights from this study and with a better understanding of the role of aortic commissures in AR, surgeons may make more informative decisions intraoperatively and continue to improve patient outcomes.

One limitation of the study is the inability to simulate the chronic adaptation that occurs with diseased tissue, as the commissure geometric alignment device was not able to recapitulate the concomitant degenerative etiology of AR. Since we used physiologically and geometrically normal aortic root tissues, our results could have somewhat deviated from the pathologic condition observed in patients with true aortic cusp prolapse. As mentioned above, the aortic cusp restriction from the fibrous band and from the support of the aortic wall likely reduced the degree of AR that could be feasibly obtained without surgically altering the aortic root geometry. Alternatively, a VSRR model could be used to investigate the impact of commissure positions without the anatomical restriction from the aortic wall and supporting tissue. However, the VSRR procedure is technically challenging and requires running sutures along the remaining aortic wall tissue. Re-implanting each commissure at a different position on the Dacron graft would require re-attaching the aortic valve to the graft each time. The aortic wall tissue would likely not be able to last for more than a few rounds of suturing, making it impossible to examine all possible permutations using the same specimen. The beauty of using the commissure geometric alignment device is that this device allows for fast and reliable repositioning of the aortic commissures without the need for suture anastomosis or valve reimplantation, therefore making it an attractive tool to investigate all ranges of commissure position using the same valve. It is important to know that this device is only able to simulate type II AR, and other etiologies of AR cannot be simulated using this device. Lastly, although porcine aortic valves were selected for this study as a human analog due to the similarities in size, anatomy, and anisotropy between porcine and human specimens [24, 25], porcine aortic cusps are thinner than that of humans [26, 27], which may also affect leaflet hemodynamics. Future investigations would need to validate findings obtained in this study using human aortic root samples. As an advancement to the current device, a new commissure geometric alignment device can be engineered to allow independent manipulation of all three aortic commissures to provide more granular results of the relationship of commissure position and AR from aortic cusp prolapse.

## Conclusions

In conclusion, we successfully designed a commissure geometric alignment device that demonstrated its ability in changing aortic commissure positions to induce aortic cusp prolapse with associated AR. We showed that AR was most prominent when commissure heights were changed from their native levels with concomitantly reduced inter-commissure angle. Findings from this study provide important evidence demonstrating the relationship between commissure position and aortic cusp prolapse. Gaining improved understanding of aortic root geometry and its relationship to aortic cusp function has a significant impact on the surgical repair of aortic valves and can help further improve patient outcomes.

## Supplementary Information

**Additional file 1**: **Table S1**. Hemodynamic Parameters for Different Commissure Height Configurations. **Table S2**: Hemodynamic Parameters for Different Inter-commissure Angle Configurations. **Table S3**: Hemodynamic Parameters for Inter-commissure Angle and Height Configurations Deviated from Baseline

## Data Availability

The datasets used and/or analyzed during the current study are available from the corresponding author on reasonable request.

## Abbreviations

AR: Aortic regurgitation
H: High commissure
HN: High commissure and narrow inter-commissure angle
HW: High commissure and wide inter-commissure angle
L: Low commissure
LN: Low commissure and narrow inter-commissure angle
LVOT: Left ventricular outflow tract
LW: Low commissure and wide inter-commissure angle
N: Narrow inter-commissure angle
VSRR: Valve-sparing aortic root replacement
W: Wide inter-commissure angle
