# Supplementary material for: Biomechanical evaluation of aortic regurgitation from cusp prolapse using an ex vivo 3D-printed commissure geometric alignment device
Source: J Cardiothorac Surg. 2022 Dec 10;17:303. doi: 10.1186/s13019-022-02049-5 (PMC9737730; doi:10.1186/s13019-022-02049-5)
Supplement: Supplementary file 1 — Additional file 1: Table S1. Hemodynamic Parameters for Different Commissure Height Configurations. Table S2: Hemodynamic Parameters for Different Inter-commissure Angle Configurations. Table S3: Hemodynamic Parameters for Inter-commissure Angle and Height Configurations Deviated from Baseline [file 13019_2022_2049_MOESM1_ESM.docx]

**SUPPLEMENTAL MATERIAL**

**Table S1**: Hemodynamic Parameters for Different Commissure Height Configurations

|  | Baseline | High Commissure | Baseline vs. High Commissure  *p* value | Low Commissure | Baseline vs. Low Commissure  *p* value |
| --- | --- | --- | --- | --- | --- |
| Heart rate (bpm) | 70.0 ± 0.0 | 70.0 ± 0.0 | - | 70.0 ± 0.0 | - |
| Systolic aortic pressure (mmHg) | 122.8 ± 1.2 | 121.8 ± 11.2 | 0.91 | 125.7± 6.6 | 0.56 |
| Diastolic aortic pressure (mmHg) | 79.7 ± 1.6 | 73.8 ± 8.7 | 0.37 | 79.0 ± 10.0 | 0.92 |
| Mean arterial pressure (mmHg) | 99.8 ± 1.0 | 96.0 ± 7.0 | 0.48 | 100.9 ± 7.1 | 0.84 |
| Cardiac output (L/min) | 5.2 ± 0.1 | 4.8 ± 0.1 | 0.04* | 5.0 ± 0.3 | 0.25 |
| Effective stroke volume (mL) | 74.5 ± 2.1 | 68.8 ± 0.9 | 0.04* | 71.9 ± 4.0 | 0.25 |
| Pump stroke volume (mL) | 109.9 ± 0.0 | 109.9 ± 0.2 | 0.94 | 109.9 ± 0.0 | 0.01* |
| Aortic forward flow time (s) | 0.3 ± 0.0 | 0.3 ± 0.0 | 0.46 | 0.3 ± 0.0 | 0.99 |
| Aortic forward flow volume (mL) | 78.1 ± 1.0 | 76.3 ± 2.9 | 0.42 | 75.0 ± 3.9 | 0.30 |
| Aortic peak forward flow rate (mL/s) | 554.6 ± 62.0 | 535.0 ± 83.5 | 0.39 | 535.0 ± 59.6 | 0.57 |
| Aortic RMS forward flow rate (mL/s) | 344.1 ± 10.6 | 289.4 ± 24.4 | 0.36 | 293.9 ± 14.6 | 0.14 |
| Aortic regurgitation fraction (%) | 4.6 ± 1.4 | 9.7 ± 3.7 | 0.26 | 4.2 ± 0.5 | 0.61 |
| Aortic leakage rate (mL/s) | -1.5 ± 1.4 | -7.3 ± 4.6 | 0.16 | -1.9 ± 1.0 | 0.47 |
| Aortic closing volume (mL) | -2.8 ± 1.7 | -4.1 ± 2.7 | 0.53 | -2.1 ± 0.8 | 0.56 |
| TransAortic forward energy loss (mJ) | 252.1 ± 120.7 | 259.0 ± 81.4 | 0.88 | 287.2 ± 134.5 | 0.80 |
| TransAortic closing energy loss (mJ) | 12.8 ± 8.1 | 18.6 ± 12.4 | 0.58 | 9.4 ± 4.8 | 0.49 |
| TransAortic leakage energy loss (mJ) | 10.4 ± 8.5 | 44.0 ± 25.7 | 0.15 | 13.3 ± 5.0 | 0.53 |
| TransAorticl total energy loss (mJ) | 275.3 ± 124.6 | 321.6 ± 58.8 | 0.52 | 309.8 ± 134.8 | 0.81 |
| Mean transaortic gradient (mmHg) | 16.3 ± 4.1 | 20.3 ± 10.7 | 0.63 | 14.0 ± 5.1 | 0.25 |

Abbreviations: RMS = root mean square.

Values are reported as mean ± standard deviation.

* denotes statistical significance with *p* < 0.05.

**Table S2**: Hemodynamic Parameters for Different Inter-commissure Angle Configurations

|  | Baseline | Narrow Inter-commissure | Baseline vs. Narrow Inter-commissure  *p* value | Wide Inter-commissure | Baseline vs. Wide Inter-commissure  *p* value |
| --- | --- | --- | --- | --- | --- |
| Heart rate (bpm) | 70.0 ± 0.0 | 70.0 ± 0.0 | - | 70.0 ± 0.0 | - |
| Systolic aortic pressure (mmHg) | 122.8 ± 1.2 | 132.6 ± 6.0 | 0.11 | 126.8± 6.5 | 0.47 |
| Diastolic aortic pressure (mmHg) | 79.7 ± 1.6 | 83.3 ± 10.2 | 0.61 | 77.6 ± 5.5 | 0.61 |
| Mean arterial pressure (mmHg) | 99.8 ± 1.0 | 106.0 ± 6.6 | 0.28 | 100.6 ± 3.8 | 0.84 |
| Cardiac output (L/min) | 5.2 ± 0.1 | 4.9 ± 0.2 | 0.08 | 5.1 ± 0.1 | 0.41 |
| Effective stroke volume (mL) | 74.5 ± 2.1 | 70.3 ± 2.8 | 0.08 | 73.5 ± 1.7 | 0.41 |
| Pump stroke volume (mL) | 109.9 ± 0.0 | 109.9 ± 0.0 | 0.76 | 109.8 ± 0.1 | 0.22 |
| Aortic forward flow time (s) | 0.3 ± 0.0 | 0.3 ± 0.0 | 0.03* | 0.3 ± 0.0 | 0.99 |
| Aortic forward flow volume (mL) | 78.1 ± 1.0 | 75.7 ± 1.1 | 0.01* | 77.5 ± 2.2 | 0.75 |
| Aortic peak forward flow rate (mL/s) | 554.6 ± 62.0 | 583.7 ± 44.2 | 0.19 | 559.3 ± 47.4 | 0.84 |
| Aortic RMS forward flow rate (mL/s) | 344.1 ± 10.6 | 310.0 ± 18.3 | 0.13 | 304.3 ± 7.0 | 0.91 |
| Aortic regurgitation fraction (%) | 4.6 ± 1.4 | 7.1 ± 3.1 | 0.33 | 5.1 ± 1.2 | 0.66 |
| Aortic leakage rate (mL/s) | -1.5 ± 1.4 | -3.7 ± 0.8 | 0.21 | -2.3 ± 0.8 | 0.30 |
| Aortic closing volume (mL) | -2.8 ± 1.7 | -3.6 ± 2.5 | 0.66 | -2.8 ± 1.5 | 0.96 |
| TransAortic forward energy loss (mJ) | 252.1 ± 120.7 | 220.5 ± 80.6 | 0.54 | 282.9 ± 92.4 | 0.51 |
| TransAortic closing energy loss (mJ) | 12.8 ± 8.1 | 16.1 ± 14.1 | 0.73 | 12.3 ± 7.9 | 0.94 |
| TransAortic leakage energy loss (mJ) | 10.4 ± 8.5 | 26.9 ± 3.7 | 0.14 | 16.4 ± 6.6 | 0.27 |
| TransAorticl total energy loss (mJ) | 275.3 ± 124.6 | 263.5 ± 78.8 | 0.78 | 311.6 ± 95.0 | 0.48 |
| Mean transaortic gradient (mmHg) | 16.3 ± 4.1 | 16.0 ± 4.5 | 0.93 | 19.3 ± 5.2 | 0.19 |

Abbreviations: RMS = root mean square.

Values are reported as mean ± standard deviation.

* denotes statistical significance with *p* < 0.05.

**Table S3**: Hemodynamic Parameters for Inter-commissure Angle and Height Configurations Deviated from Baseline

|  | HW | Baseline vs. HW  *p* value | HN | Baseline vs. HN  *p* value | LW | Baseline vs. LW  *p* value | LN | Baseline vs. LN  *p* value |
| --- | --- | --- | --- | --- | --- | --- | --- | --- |
| Heart rate (bpm) | 70.0 ± 0.0 | - | 70.0 ± 0.0 | - | 70.0 ± 0.0 | - | 70.0 ± 0.0 | - |
| Systolic aortic pressure (mmHg) | 113.1 ± 19.3 | 0.53 | 110.2 ± 9.0 | 0.20 | 122.9± 6.1 | 0.98 | 126.7 ± 4.0 | 0.27 |
| Diastolic aortic pressure (mmHg) | 68.5 ± 6.3 | 0.11 | 66.1 ± 10.2 | 0.16 | 76.6 ± 8.2 | 0.57 | 77.5 ± 11.1 | 0.78 |
| Mean arterial pressure (mmHg) | 88.2 ± 11.5 | 0.27 | 85.6 ± 8.7 | 0.15 | 97.7 ± 5.4 | 0.61 | 100.3 ± 6.9 | 0.92 |
| Cardiac output (L/min) | 4.6 ± 0.3 | 0.05* | 4.7 ± 0.6 | 0.29 | 5.2 ± 0.1 | 0.34 | 5.0 ± 0.2 | 0.17 |
| Effective stroke volume (mL) | 66.4 ± 4.3 | 0.05* | 67.3 ± 9.2 | 0.29 | 74.1 ± 1.8 | 0.34 | 71.4 ± 2.5 | 0.17 |
| Pump stroke volume (mL) | 109.8 ± 0.1 | 0.46 | 109.8 ± 0.1 | 0.12 | 109.9 ± 0.0 | 0.29 | 109.8 ± 0.1 | 0.33 |
| Aortic forward flow time (s) | 0.3 ± 0.0 | 0.91 | 0.3 ± 0.0 | 0.87 | 0.3 ± 0.0 | 0.22 | 0.3 ± 0.0 | 0.24 |
| Aortic forward flow volume (mL) | 75.2 ± 2.9 | 0.25 | 77.3 ± 5.7 | 0.86 | 77.8 ± 1.2 | 0.36 | 77.0 ± 2.2 | 0.36 |
| Aortic peak forward flow rate (mL/s) | 466.3 ± 94.8 | 0.06 | 492.3 ± 72.0 | 0.12 | 516.3 ± 77.3 | 0.17 | 524.5 ± 33.1 | 0.54 |
| Aortic RMS forward flow rate (mL/s) | 281.8 ± 34.7 | 0.39 | 292.5 ± 23.3 | 0.43 | 297.4 ± 20.7 | 0.02* | 305.8 ± 17.7 | 0.98 |
| Aortic regurgitation fraction (%) | 11.7 ± 5.8 | 0.21 | 13.0 ± 8.5 | 0.26 | 4.8 ± 0.9 | 0.67 | 7.3 ± 1.7 | 0.28 |
| Aortic leakage rate (mL/s) | -4.9 ± 2.3 | 0.32 | -5.0 ± 2.8 | 0.29 | -1.7 ± 3.1 | 0.91 | -4.9 ± 1.6 | 0.002* |
| Aortic closing volume (mL) | -6.5 ± 3,9 | 0.29 | -7.6 ± 6.7 | 0.36 | -2.9 ± 1.3 | 0.95 | -3.2 ± 1.8 | 0.81 |
| TransAortic forward energy loss (mJ) | 433.5 ± 122.1 | 0.01* | 329.5 ± 57.7 | 0.24 | 444.5 ± 177.3 | 0.06 | 369.9 ± 89.3 | 0.33 |
| TransAortic closing energy loss (mJ) | 39.6 ± 24.7 | 0.26 | 35.9 ± 32.2 | 0.37 | 14.9 ± 10.1 | 0.76 | 15.4 ± 10.8 | 0.77 |
| TransAortic leakage energy loss (mJ) | 26.7 ± 12.5 | 0.38 | 27.8 ± 17.4 | 0.36 | 11.7 ± 18.7 | 0.92 | 32.4 ± 9.2 | 0.01* |
| TransAorticl total energy loss (mJ) | 499.8 ± 132.2 | 0.04* | 393.2 ± 76.2 | 0.09 | 471.1 ± 187.2 | 0.07 | 417.7 ± 90.0 | 0.28 |
| Mean transaortic gradient (mmHg) | 25.7 ± 10.8 | 0.31 | 17.7 ± 3.7 | 0.63 | 21.0 ± 7.5 | 0.34 | 21.3 ± 2.6 | 0.24 |

Abbreviations: HW = high commissure and wide inter-commissure angle, HN = high commissure and narrow inter-commissure angle, LW = low commissure and wide inter-commissure angle, LN = low commissure and narrow inter-commissure angle, RMS = root mean square.

Values are reported as mean ± standard deviation.

* denotes statistical significance with *p* < 0.05.
